# Supplementary material for: Human TRPV1 structure and inhibition by the analgesic SB-366791
Source: Nat Commun. 2023 Apr 28;14:2451. doi: 10.1038/s41467-023-38162-9 (PMC10147690; doi:10.1038/s41467-023-38162-9)
Supplement: Supplementary file 1 — Supplementary Information [file 41467_2023_38162_MOESM1_ESM.pdf]

Supplementary Information

Human TRPV1 structure and inhibition by the analgesic SB-366791

Arthur Neuberger[1], Mai Oda[2], Yury A. Nikolaev[2], Kirill D. Nadezhdin[1], Elena O. Gracheva[2,3,4,5], Sviatoslav N. Bagriantsev[2], Alexander I. Sobolevsky[1,*]

[1] Department of Biochemistry and Molecular Biophysics, Columbia University, New York, NY, USA

[2] Department of Cellular and Molecular Physiology, Yale University School of Medicine, New Haven, CT 06510, USA.

[3] Department of Neuroscience, Yale University School of Medicine, New Haven, CT 06510, USA.

[4] Program in Cellular Neuroscience, Neurodegeneration and Repair, Yale University School of Medicine, New Haven, CT 06510, USA.

[5] Kavli Institute for Neuroscience, Yale University School of Medicine, New Haven, CT 06510, USA.

*Corresponding author. Tel: +1 2123054249; E-mail: as4005@cumc.columbia.edu

This PDF file includes:

Supplementary Figures 1-7

Supplementary Table 1

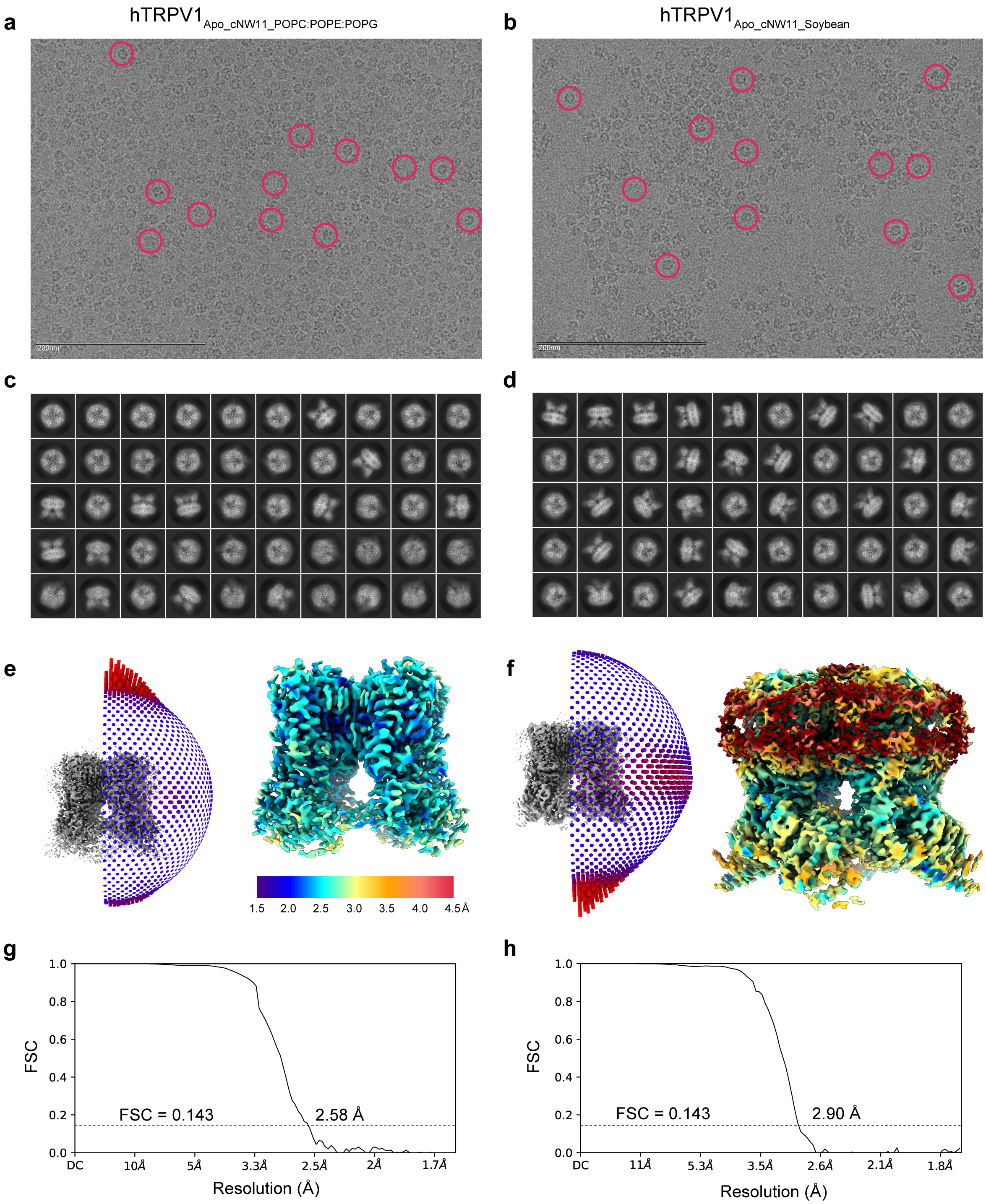

Supplementary Fig. 1 | Overview of cryo-EM data for hTRPV1$_{Apo}$. a, b Representatives of 16,062 and 19,303 micrographs for hTRPV1$_{Apo}$ in synthetic (**a**) and soybean (**b**) lipids, respectively, with example particles circled in pink. **c, d** Representative 2D class averages for hTRPV1$_{Apo}$ in synthetic (**c**) and soybean (**d**) lipids. **e, f** Euler angle distribution of particles contributing to final reconstructions with larger red cylinders representing orientations comprising more particles (left) and local resolution presented as coloring of the map (right) for hTRPV1$_{Apo}$ in synthetic (**e**) and soybean (**f**) lipids. **g, h** FSC curves for hTRPV1$_{Apo}$ in synthetic (**g**) and soybean (**h**) lipids.

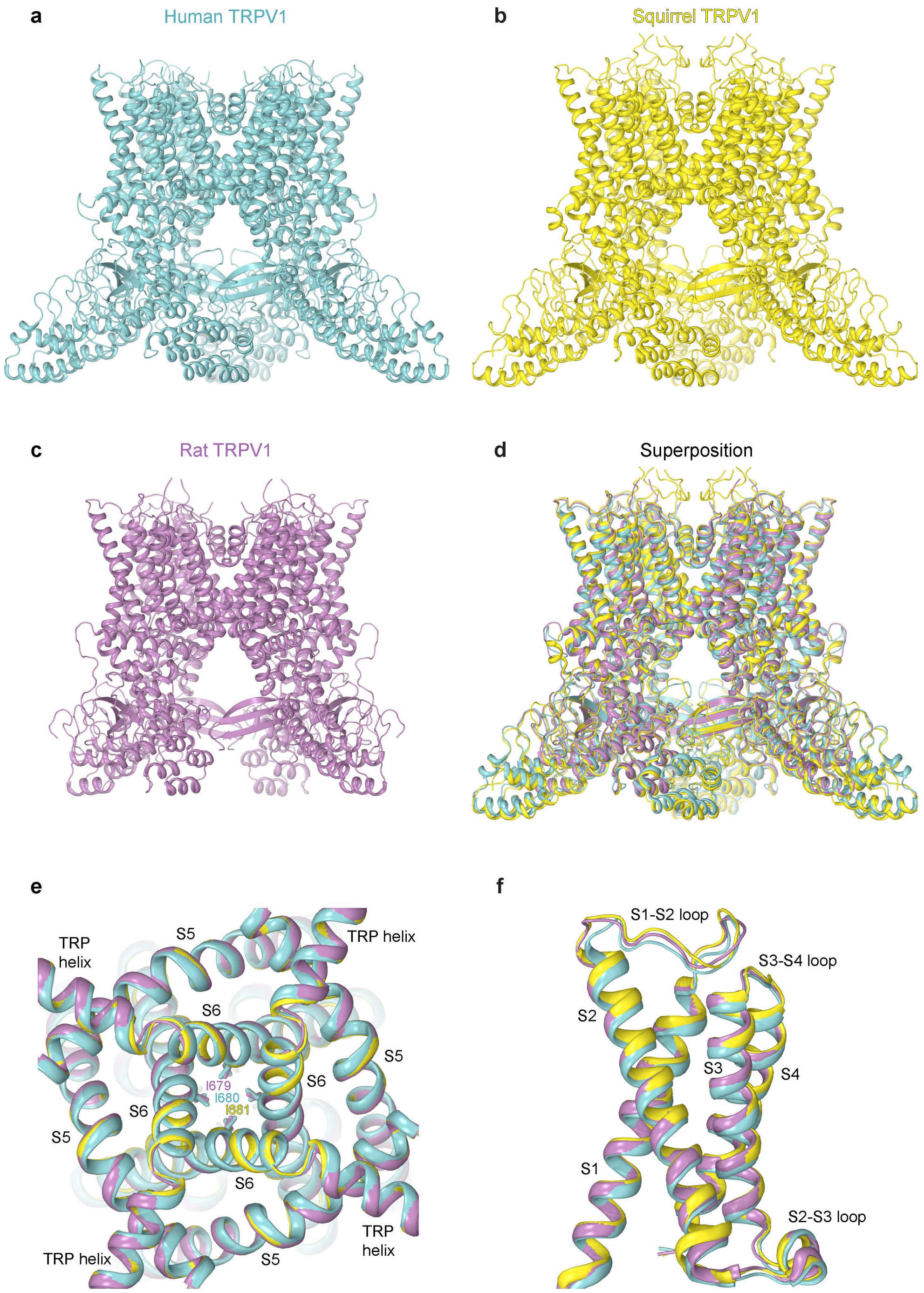

Supplementary Fig. 2 | Comparison of human, squirrel, and rat TRPV1 structures. a-c Apo-state cryo-EM structures of human (**a**, cyan), squirrel (**b**, yellow, PDB ID: 7LQY) and rat (**c**, purple, PDB ID: 7L2H) TRPV1 viewed parallel to the membrane. **d** Superposition of structures shown in **a-c**. **e**, **f** Close-up views of the pore (**e**) and S1-S4 (**f**) domains in structures superposed in **d**.

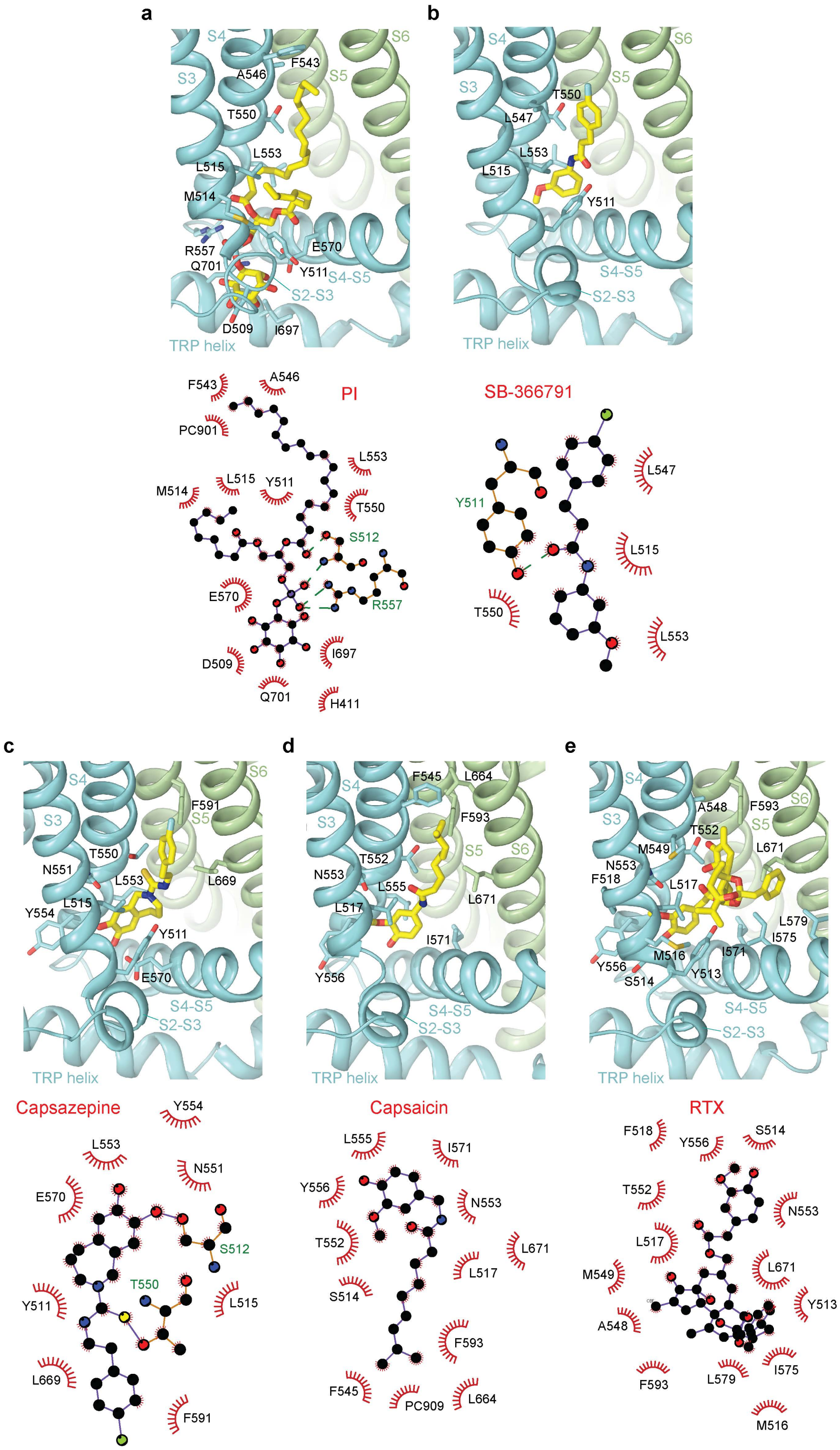

Supplementary Fig. 3 | Vanilloid site ligands of TRPV1. a-e Close-up views (top) and LigPlot analysis (bottom) of the vanilloid site in human TRPV1 bound to PI (**a**, $hTRPV1_{Apo}$), human TRPV1 bound to SB-366791 (**b**, $hTRPV1_{SB\text{-}366791}$), rat TRPV1 bound to capsazepine (**c**, PDB ID: 5IS0), squirrel TRPV1 bound to capsaicin (**d**, PDB ID: 7LR0) and squirrel TRPV1 bound to RTX (**e**, PDB ID: 7LQZ). In the close-up views, the molecules of phosphatidylinositol, SB-366791, capsazepine, capsaicin and RTX, and residues that contribute to their binding are shown in sticks.

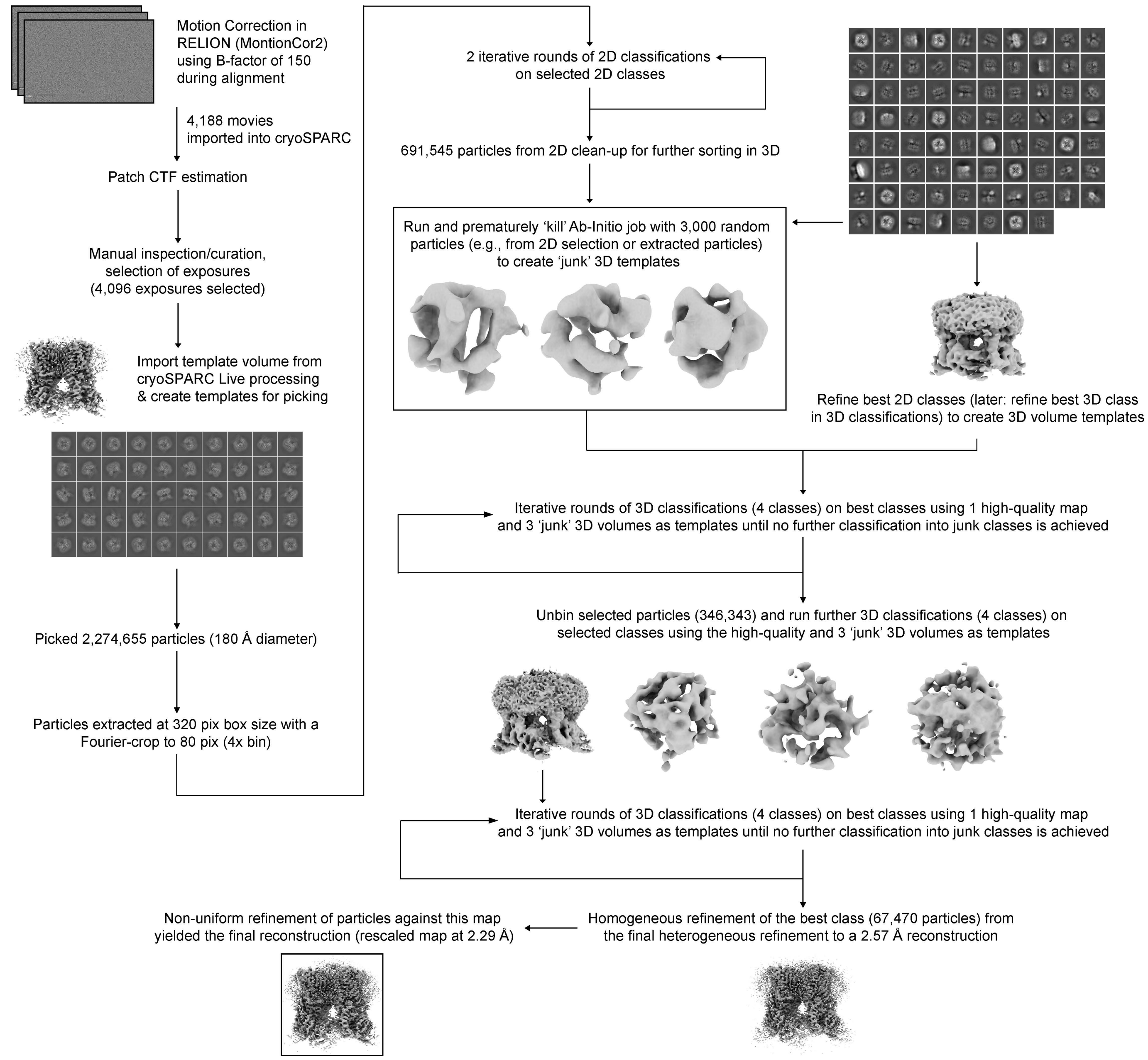

Supplementary Fig. 4 | Representative 3D reconstruction workflow. 3D reconstruction workflow is shown for $TRPV1_{SB\text{-}366791}$. A similar workflow was used for the $TRPV1_{Apo}$ structures.

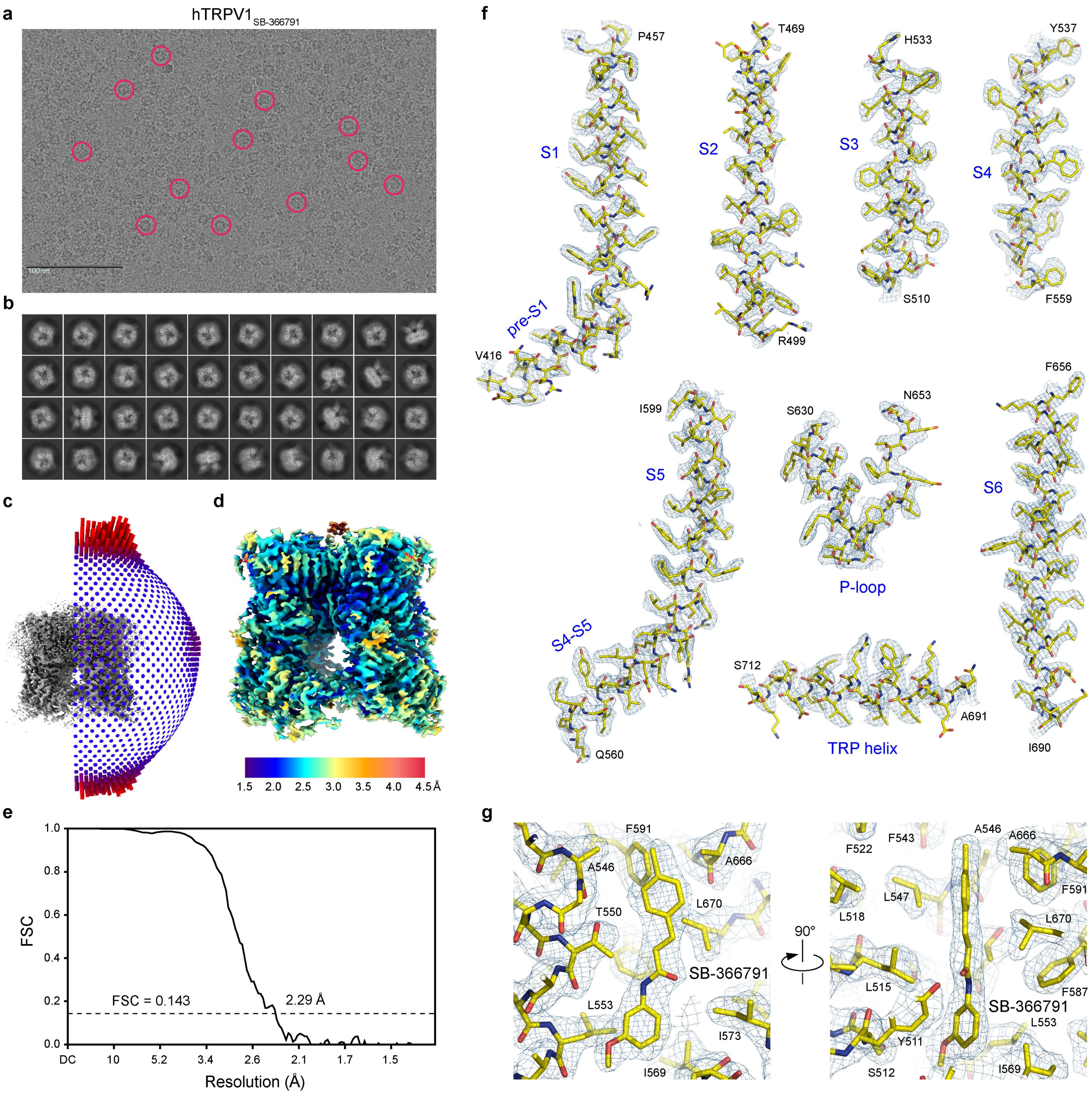

Supplementary Fig. 5 | Overview of cryo-EM data for TRPV1$_{SB\text{-}366791}$. **a** Representative of 4,188 micrographs for hTRPV1$_{SB\text{-}366791}$, with example particles circled in pink. **b** Representative 2D class averages. **c** Euler angle distribution of particles contributing to the final reconstruction with larger red cylinders representing orientations comprising more particles. **d** Local resolution presented as coloring of the hTRPV1$_{SB\text{-}366791}$ map. **e** FSC curve calculated between half maps, with the resolution range estimated using the FSC = 0.143 criterion. **f** Fragments of the 2.29-Å resolution hTRPV1$_{SB\text{-}366791}$ cryo-EM map (blue mesh) for the membrane segments and TRP helix. **g** Binding site of SB-366791, with the inhibitor and protein shown in sticks and cryo-EM map as a blue mesh.

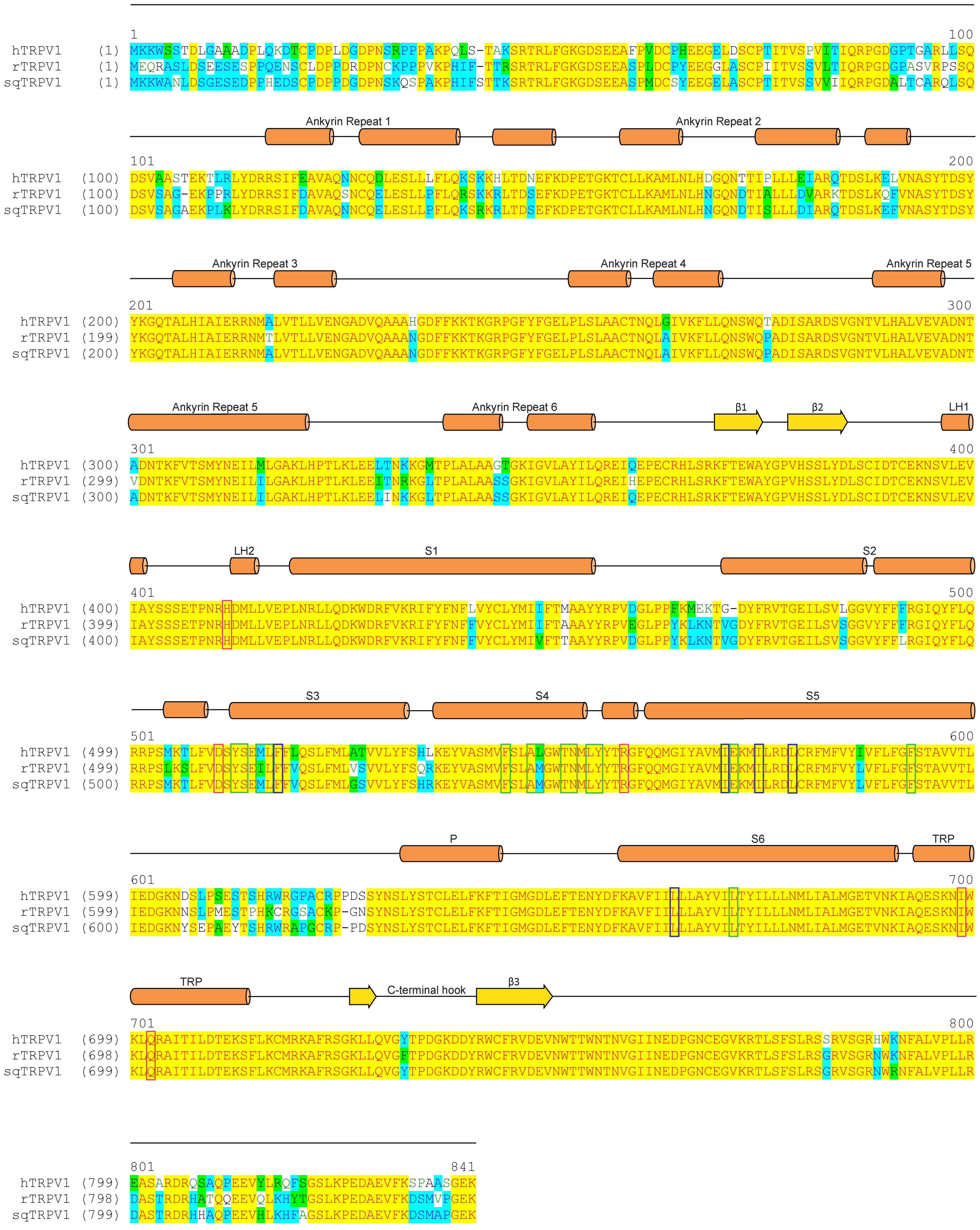

Supplementary Fig. 6 | Sequence alignment of human, rat and squirrel TRPV1. α helices and β strands are depicted above the sequences as cylinders and arrows, respectively. Boxes indicate residues involved in binding of vanilloid-site antagonists (red), agonists (blue) or both (green).

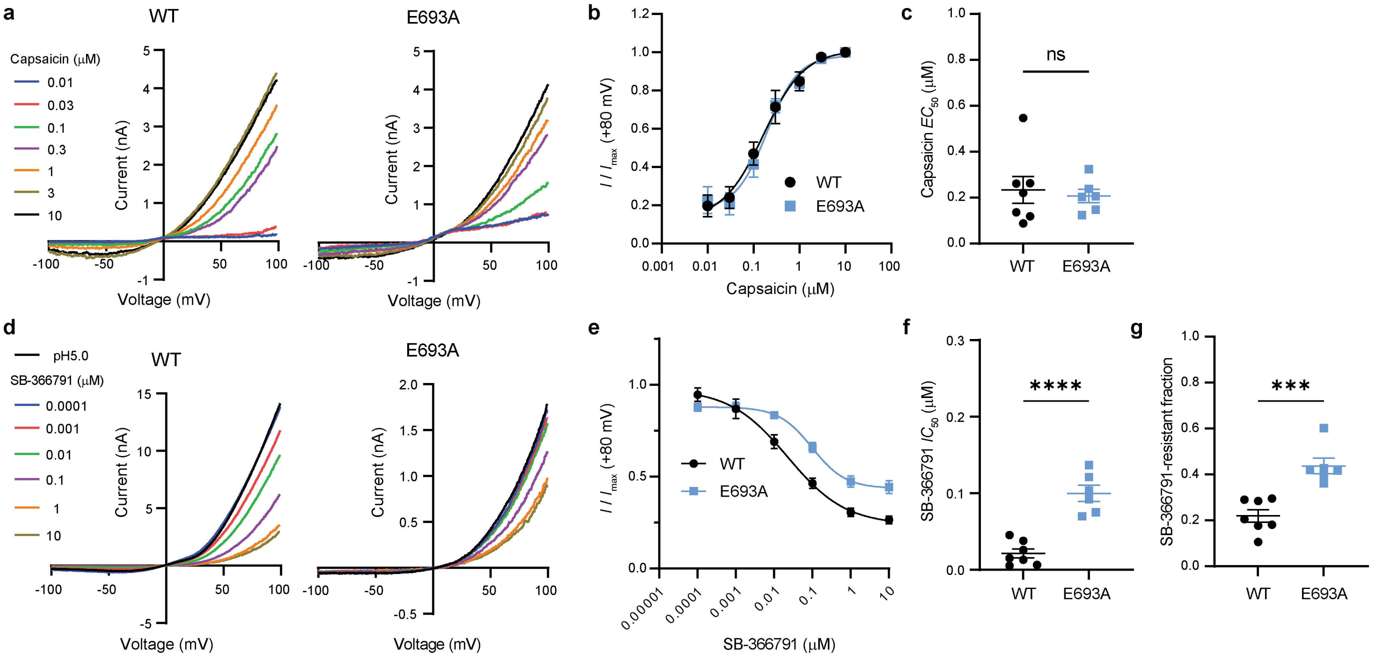

Supplementary Fig. 7 | Functional characterization of the E693A mutant. a Exemplar whole-cell current traces evoked by a voltage ramp in HEK 293 cells expressing hTRPV1-wild type and E693A mutant in the presence of capsaicin from 0.01 to 10 μM. **b** Concentration-dependence of hTRPV1 wild-type and E693A mutant activation by capsaicin measured at +80 mV. Data are shown as mean ± SEM. Lines represent fits to the Hill equation (WT, n = 7; E693A, n = 6). **c** Quantification of half-maximal effective capsaicin concentration (EC_{50}) for hTRPV1-wild type and E693A mutant. Data are shown as mean ± SEM. Points represent EC_{50} estimates from Hill fits of recordings from individual cells. (WT, n = 7; E693A, n = 6). Statistical analysis: two-sided *t*-test. $P = 0.7099$. **d** Exemplar whole-cell currents recorded from hTRPV1-wild type and E693A mutant in HEK 293 cells at pH 5 in response to a voltage ramp in the presence of different concentrations of SB-366791. **e** Concentration-dependence of wild-type and E693A mutant hTRPV1 inhibition by SB-366791 measured at +80 mV. Data are shown as mean ± SEM. Lines represent fits to the Hill equation (WT, n = 7; E693A, n = 6). **f** Quantification of half-maximal inhibitory concentration (IC_{50}) of SB-366791 for hTRPV1-wild type and E693A mutant. Data are shown as mean ± SEM. Points represent IC_{50} estimates from Hill fits of recordings from individual cells. (WT, n = 7; E693A, n = 6). Statistical analysis: two-sided *t*-test. ****$P < 0.0001$. **g** Quantification of SB-366791-resistant current for hTRPV1-wild type and E693A mutant. Data are shown as mean ± SEM. Points represent minimal current estimates from Hill fits of recordings from individual cells. (WT, n = 7; E693A, n = 6). Statistical analysis: two-sided *t*-test. ***$P = 0.0004$. Source data are provided as a Source Data file.

Supplementary Table 1 | Cryo-EM data collection, refinement, and validation statistics

**Structure**	$hTRPV1_{Apo}$	$hTRPV1_{Apo}$	$hTRPV1_{SB\text{-}366791}$
Preparation	cNW11/soybean lipids	cNW11/synthetic lipids	GDN
EMDB accession code	EMD-29981	EMD-29982	EMD-29983
PDB accession code	8GF8	8GF9	8GFA
**Data collection and processing**			
Magnification	105,000x	47,000x	130,000x
Voltage (kV)	300	300	300
Electron exposure ($e^{-}Å^{-2}$)	50	60	60
Defocus range (μm)	−0.75 to −2.0	−0.8 to −2.0	−0.75 to −1.5
Reported pixel size (Å)	0.825	0.785	0.645
Exposures (no.)	19,303	16,062	4,188
**Processing software**			
Motion correction	cryoSPARC v3.3	cryoSPARC v3.3	RELION v4.0
CTF estimation	cryoSPARC v3.3	cryoSPARC v3.3	cryoSPARC v3.3
Platform software for particle picking	cryoSPARC v3.3	cryoSPARC v3.3	cryoSPARC v3.3
Software for 2D/3D class. & Refinements	cryoSPARC v3.3	cryoSPARC v3.3	cryoSPARC v3.3
Symmetry imposed	C4	C4	C4
Initial particle images (no.)	8,994,098	6,558,023	1,988,636
Final particle images (no.)	323,292	778,428	67,470
Map resolution (Å) FSC 0.143	2.90	2.58	2.29
**Refinement**			
Initial models used (PDB code)	7LQY	7LQY	7LQY
Model resolution (Å)	2.90	2.58	2.29
FSC threshold	0.143	0.143	0.143
Map sharpening *B* factor ($Å^2$)	−159.2	−110.6	−84.6
**Model composition**			
Non-hydrogen atoms	22,282	19,290	19,323
Protein residues	2,520	2,092	2,124
Ligands	42	46	46
Water	0	92	105
B factors ($Å^2$)			
Protein	64.44	79.85	62.31
Ligands	34.36	24.61	28.95
Water	n.a.	17.94	14.01
R.m.s. deviations			
Bond lengths (Å)	0.010	0.010	0.013
Bond angles (°)	1.320	1.359	1.420
**Validation**			
MolProbity score	1.66	1.51	1.53
Clash score, all atoms	2.75	2.84	3.21
Poor rotamers (%)	0.00	0.00	0.00
Ramachandran plot			
Favored (%)	88.98	94.00	94.69
Allowed (%)	10.70	5.80	5.12
Disallowed (%)	0.32	0.19	0.19
