# Supplementary material for: Human TRPV1 structure and inhibition by the analgesic SB-366791
Source: Nat Commun. 2023 Apr 28;14:2451. doi: 10.1038/s41467-023-38162-9 (PMC10147690; doi:10.1038/s41467-023-38162-9)
Supplement: Supplementary file 3 — Reporting Summary [file 41467_2023_38162_MOESM3_ESM.pdf]

nature portfolio

Corresponding author(s): Alexander Sobolevsky
Last updated by author(s): Mar 30, 2023

Reporting Summary

Nature Portfolio wishes to improve the reproducibility of the work that we publish. This form provides structure for consistency and transparency in reporting. For further information on Nature Portfolio policies, see our Editorial Policies and the Editorial Policy Checklist.

Statistics

For all statistical analyses, confirm that the following items are present in the figure legend, table legend, main text, or Methods section.

n/a	Confirmed	
☐	☒	The exact sample size (*n*) for each experimental group/condition, given as a discrete number and unit of measurement
☐	☒	A statement on whether measurements were taken from distinct samples or whether the same sample was measured repeatedly
☐	☒	The statistical test(s) used AND whether they are one- or two-sided<br>*Only common tests should be described solely by name; describe more complex techniques in the Methods section.*
☒	☐	A description of all covariates tested
☒	☐	A description of any assumptions or corrections, such as tests of normality and adjustment for multiple comparisons
☐	☒	A full description of the statistical parameters including central tendency (e.g. means) or other basic estimates (e.g. regression coefficient) AND variation (e.g. standard deviation) or associated estimates of uncertainty (e.g. confidence intervals)
☐	☒	For null hypothesis testing, the test statistic (e.g. *F*, *t*, *r*) with confidence intervals, effect sizes, degrees of freedom and *P* value noted<br>*Give P values as exact values whenever suitable.*
☒	☐	For Bayesian analysis, information on the choice of priors and Markov chain Monte Carlo settings
☒	☐	For hierarchical and complex designs, identification of the appropriate level for tests and full reporting of outcomes
☒	☐	Estimates of effect sizes (e.g. Cohen's *d*, Pearson's *r*), indicating how they were calculated

Our web collection on statistics for biologists contains articles on many of the points above.

Software and code

Policy information about availability of computer code

Data collection	pCLAMP 10.3, EPU 2, SerialEM 4.0
Data analysis	MotionCor2, cryoSPARC 3.3, UCSF Chimera 1.16, UCSF ChimeraX 1.3, COOT 0.9.8.1, PHENIX 1.19.2, PyMOL 2.5.2, HOLE 2.1

For manuscripts utilizing custom algorithms or software that are central to the research but not yet described in published literature, software must be made available to editors and reviewers. We strongly encourage code deposition in a community repository (e.g. GitHub). See the Nature Portfolio guidelines for submitting code & software for further information.

Data

Policy information about availability of data

All manuscripts must include a data availability statement. This statement should provide the following information, where applicable:

- Accession codes, unique identifiers, or web links for publicly available datasets
- A description of any restrictions on data availability
- For clinical datasets or third party data, please ensure that the statement adheres to our policy

All data needed to evaluate the conclusions of the paper are present in the paper or the Supplementary Information. The cryo-EM density maps of hTRPV1 in apo-states and in complex with SB-366791 were deposited to the Electron Microscopy Data Bank (EMDB) under the accession codes EMD-29981 (hTRPV1Apo; cNW11, soybean lipids, https://www.emdataresource.org/EMD-29981), EMD-29982 (hTRPV1Apo; cNW11, POPC:POPE:POPG, https://www.emdataresource.org/EMD-29982), and EMD-29983 (hTRPV1SB-366791, https://www.emdataresource.org/EMD-29983), respectively.
The atomic coordinates have been deposited to the Protein Data Bank (PDB) under the accession codes 8GF8 (hTRPV1Apo; cNW11/soybean lipids, https://www.rcsb.org/structure/unreleased/8GF8), 8GF9 (hTRPV1Apo; cNW11/POPC:POPE:POPG, https://www.rcsb.org/structure/unreleased/8GF9), and 8GFA

(hTRPV1SB-366791, https://www.rcsb.org/structure/unreleased/8GFA) (see Supplementary Table 1), respectively. All other data are available from the corresponding author upon request. Source data are provided with this paper.

Field-specific reporting

Please select the one below that is the best fit for your research. If you are not sure, read the appropriate sections before making your selection.

☒ Life sciences ☐ Behavioural & social sciences ☐ Ecological, evolutionary & environmental sciences

For a reference copy of the document with all sections, see nature.com/documents/nr-reporting-summary-flat.pdf

Life sciences study design

All studies must disclose on these points even when the disclosure is negative.

Sample size	Amount of cryo-EM data collected was limited by time allocation at the microscopes. For patch-clamp experiments, no sample size predetermination was performed. The reported sample size is based on accepted methodology and published works by us and others, and is sufficient to obtain reproducible and reliable data from patch-clamp experiments in HEK cells using TRPV channels.
Data exclusions	No data has been excluded.
Replication	No replication attempts have failed. The cryo-EM data collection was consistent from the beginning to the end. In patch-clamp studies, at least three independent replicates with independent cellular preparations were performed for each experiment.
Randomization	Samples were not randomized; it is not technically or practically feasible to do so for cryo-EM or patch-clamp electrophysiological experiments. Ranadomization is inherent to patch-clamp experiments, because the experimenter picks cells from the coverslip at random.
Blinding	Researchers were not blinded; it is not technically or practically feasible to do so for cryo-EM or patch-clamp electrophysiological experiments.

Reporting for specific materials, systems and methods

We require information from authors about some types of materials, experimental systems and methods used in many studies. Here, indicate whether each material, system or method listed is relevant to your study. If you are not sure if a list item applies to your research, read the appropriate section before selecting a response.

Materials & experimental systems

n/a	Involved in the study	
☒	☐	Antibodies
☐	☒	Eukaryotic cell lines
☒	☐	Palaeontology and archaeology
☒	☐	Animals and other organisms
☒	☐	Human research participants
☒	☐	Clinical data
☒	☐	Dual use research of concern

Methods

n/a	Involved in the study	
☒	☐	ChIP-seq
☒	☐	Flow cytometry
☒	☐	MRI-based neuroimaging

Eukaryotic cell lines

Policy information about cell lines

Cell line source(s)	HEK293S GnTI-, ATCC, Cat#CRL-3022<br>HEK293, ATCC, Cat#CRL-1573<br>Sf9, Gibco, Cat#12659017<br>HEK293TΔPIEZO1, Dr. Ardem Patapoutian (Scripps Research Institute) (Lukacs et al., 2015)
Authentication	None of the cell lines used have been authenticated.
Mycoplasma contamination	The cell lines used have been tested for mycoplasma contamination by the providers (negative results) but have not been retested in the lab.
Commonly misidentified lines (See ICLAC register)	No commonly misidentified lines were used in this study.
